# Hemophagocytic Lymphohistiocytosis Triggered by *Legionella pneumophila* and SARS‐CoV‐2 Infection in GATA2 Deficiency

**DOI:** 10.1002/ccr3.72699

**Published:** 2026-05-12

**Authors:** Harry Wilson, Nuno Borges, Matthew Collin, Callum Wright

**Affiliations:** ^1^ Haematopoiesis and Immunity Laboratory Newcastle University Newcastle upon Tyne UK

**Keywords:** hematology, immunology, infectious diseases, oncology, transplantation

## Abstract

Germline GATA2 deficiency predisposes to bone marrow failure, myeloid neoplasia, and immune dysregulation. The syndrome is often complicated by infection with intracellular pathogens and viruses, autoimmunity, and inflammation. Hemophagocytic lymphohistiocytosis (HLH) is a rare occurrence that can present further management challenges. Here, we describe a young adult with GATA2 deficiency presenting with *Legionella* pneumonia, COVID‐19, and HLH with underlying *SF3B1*‐mutated myelodysplasia that responded successfully to allogeneic hematopoietic stem cell transplantation.

## Key Clinical Message

GATA2 deficiency should be excluded in all young adults presenting with atypical infection and bone marrow failure, and it is an uncommon but recognized cause of HLH. Allograft should be considered in eligible patients with life‐threatening complications of immunodeficiency, immune dysregulation, or acquisition of secondary clonal mutations in the bone marrow.

## 1 Introduction

Hemophagocytic lymphohistiocytosis (HLH) is a life‐threatening condition characterized by systemic hyperinflammation secondary to cytokine storm and the pathological activation of T cells and macrophages [1, 2]. The condition has traditionally been categorized as either primary HLH, a hereditary disease, or secondary HLH, triggered by acquired pathology such as infection or malignancy [2]. More contemporary conceptualizations of HLH recognize a continuum of disease influenced by both predisposing genetic factors and environmental triggers [1].

HLH is exceedingly rare; its incidence in people aged ≥ 15 years between 2000 and 2016 in England has recently been estimated at 1.3 per million person years [3]. The three cardinal features of refractory fever, hyperferritinaemia, and cytopenia should trigger a rapid assessment by the HScore, which uses commonly available tests, or the optimized HLH inflammatory index, which incorporates ferritin and sCD25 and has proven value in malignancy‐associated HLH in adults [4, 5]. Untreated HLH is rapidly fatal, and early diagnosis is key [6]. A rising incidence across multiple countries suggests that recognition is improving [7, 8].

The management of HLH in adults involves the administration of corticosteroids (with or without intravenous immunoglobulin) and the identification and prompt treatment of underlying triggers [6]. When an inherited genetic abnormality is thought to have caused HLH, allogeneic hematopoietic stem cell transplantation (allo‐HSCT) may be required to prevent recurrent episodes, at the risk of significant treatment‐related morbidity and mortality [6, 9, 10]. Survival in HLH is variable and is influenced by age, sex, and underlying cause [3]. Underlying hematological malignancy appears to be a negative prognostic factor [3].

GATA2 deficiency syndrome (G2DS) is one of the most common constitutional genetic causes of bone marrow failure, often presenting with infection due to the failure of mononuclear cell production and profound monocytopenia, typically causing defective immunity to intracellular pathogens [11]. G2DS represents a spectrum of disease caused by heterozygous germline mutations in the *GATA2* gene, which encodes a zinc finger transcription factor fulfilling important roles in maintaining a pool of hematopoietic stem cells and facilitating progenitor cell differentiation [12, 13]. These mutations can either be inherited or occur de novo, and many pathogenic variants have been identified [14, 15]. Truncating mutations throughout *GATA2*, regulatory mutations, and missense mutations affecting the zinc finger 2 (ZF2) domain are particularly well described [14, 15]. The clinical phenotype of G2DS is highly variable but includes an immunodeficiency syndrome characterized by a deficiency in dendritic cells, monocytes, B cells, and NK cells, as well as myeloid neoplasms, such as myelodysplastic neoplasm (MDS) and acute myeloid leukemia (AML) [11, 12]. Other complications include Emberger syndrome, autoimmunity, viral‐driven neoplasia, pulmonary dysfunction, and deep vein thrombosis (DVT) [11, 16]. In 2021, ten years after G2DS was first reported, a review noted that over 480 individuals with a pathogenic GATA2 deficiency had been documented [14, 17, 18].

Germline defects should be suspected in children and young adults presenting with MDS, which has a median age of onset of 12–34 years in G2DS [15, 16, 19]. In a study of 106 patients with G2DS‐associated myeloid malignancies, somatic mutations in *ASXL1* and *STAG2* were the most frequent additional variants identified; however, other mutations commonly seen in MDS were not present in this cohort [20]. *SF3B1* encodes a splicing factor frequently associated with myeloid malignancies, leading to a specific MDS phenotype consisting of an excess of ring sideroblasts in the bone marrow, earning its own entry in the World Health Organization Classification of Hematolymphoid Tumors [21]. *SF3B1* mutations are seen in over 20% of MDS cases but have not been previously reported in GATA2 deficiency [20, 22].

Predicting prognosis in G2DS is particularly challenging, as some patients will develop life‐threatening complications at a young age, and others remain asymptomatic for many decades [12]. Diagnosis is achieved through genetic testing, and the only curative treatment option is allo‐HSCT [15]. Treatment is typically reserved for the onset of life‐threatening complications such as MDS, AML, severe infection, and viral‐driven neoplasia [15]. Promptly recognizing G2DS when it is present in patients presenting with HLH is vital, as it represents a potentially curable underlying cause, and correcting genetic susceptibility with allo‐HSCT maximizes the likelihood of achieving sustained HLH remission [12, 15, 23, 24].

HLH is a rare presentation of G2DS; a 2024 systematic review identified only 23 reported cases across 15 publications, and no new cases have been identified since [23]. The mean age of onset was 23 years, and *GATA2* mutations among first‐degree relatives of patients were common. A diverse array of HLH‐triggering infections was reported in this patient group, including *Mycobacterium avium, Mycobacterium kansasii*, influenza A, Epstein–Barr virus (EBV), varicella‐zoster virus (VZV), cytomegalovirus (CMV), and herpes simplex virus (HSV). Underlying *GATA2* mutations appeared equally heterogeneous. When compared with non‐G2DS HLH cohorts, this group had a lower age at onset, greater female predominance, higher rates of EBV, CMV, and VZV infection, and more frequent underlying hematological malignancies [3, 7]. Mortality appeared broadly comparable to HLH at large [3]. No published estimates of the incidence of HLH in patients with G2DS or the prevalence of G2DS in patients presenting with HLH exist. Using available data, HLH appears to occur in < 5% of G2DS cases, although this estimate may be inaccurate due to the potential underdiagnosis of both conditions [1, 14, 15, 23]. In this report, we present a further case of HLH in a patient with undiagnosed G2DS, which is unique for an associated *SF3B1‐*mutated MDS.

## 2 Presentation, History, and Examination

A 25‐year‐old male presented to the emergency department (ED) following a collapse. Review of systems revealed a four‐month history of fatigue and a three‐day history of nausea, malaise, and anorexia. Past medical history was unremarkable, and family history was positive only for an unspecified paternal malignancy at approximately age 60. Clinical examination noted sinus tachycardia, marked pallor, and pyrexia (38.8°C). A full blood count revealed a hemoglobin of 29 g/L (normal range, NR: 130–180 g/L), reticulocyte count of 16.3 × 10^9^/L (NR: 50–100 × 10^9^/L), white cell count of 2.07 × 10^9^/L (NR: 4–11 × 10^9^/L), neutrophil count of 1.22 × 10^9^/L (NR: 2–7 × 10^9^/L), monocyte count of 0.14 × 10^9^/L (NR: 0.2–0.8 × 10^9^/L), lymphocyte count of 0.7 × 10^9^/L (NR: 1–4.5 × 10^9^/L) and platelet count of 828 × 10^9^/L (NR: 150–450 × 10^9^/L). C‐reactive protein was 192 mg/L (NR: 0–5 mg/L). Alanine transaminase was 1521 U/L (NR: 0–40 U/L), and aspartate transaminase was 797 U/L (NR: 0–50 U/L). Two units of packed red blood cells were transfused; empirical antibiotic therapy with piperacillin and tazobactam was commenced, and the patient was admitted to the hematology ward for further investigation and management.

## 3 Differential Diagnosis

The differential diagnosis of the initial history and clinical findings, combined with profound anemia, reticulocytopenia, neutropenia, deranged liver function tests, and raised inflammatory markers, was broad—including HLH, acute leukemia, high‐grade lymphoma, aplastic anemia, acute hepatitis, red cell aplasia (e.g., secondary to parvovirus B19 infection) or other severe acute viral infections.

## 4 Initial Investigation and Treatment

The results of blood tests requested on admission are reported in Table 1. Hyperferritinaemia, raised lactate dehydrogenase, raised vitamin B12, raised Clauss fibrinogen, and mildly raised troponin T were noted, in addition to the results already available in ED. Folate and triglycerides were within normal range.

**TABLE 1:** Notable results from a peripheral venous blood sample taken on admission.

Test	Normal Range	Result	Units
Hemoglobin	130–180	29	g/L
Platelets	150–450	828	× 10^9^/L
White Cells	4–11	2.07	× 10^9^/L
Neutrophils	2–7	1.22	× 10^9^/L
Lymphocytes	1–4.5	0.7	× 10^9^/L
Monocytes	0.2–0.8	0.14	× 10^9^/L
Reticulocytes	50–100	16.3	× 10^9^/L
Vitamin B12	145–569	1,476	pmol/L
Folate	3.89–26.8	11.7	μg/L
C‐reactive Protein	0–5	192	mg/L
Alanine Transaminase	0–40	1521	U/L
Aspartate Transaminase	0–50	797	U/L
Lactate Dehydrogenase	135–225	2,033	U/L
Ferritin	60–300	21,944	μg/L
Troponin T	0–11	49	ng/L
Triglycerides	0.5–1.7	0.8	mmol/L
Clauss Fibrinogen	2.1–4.8	7.5	g/L

**FIGURE 1:**
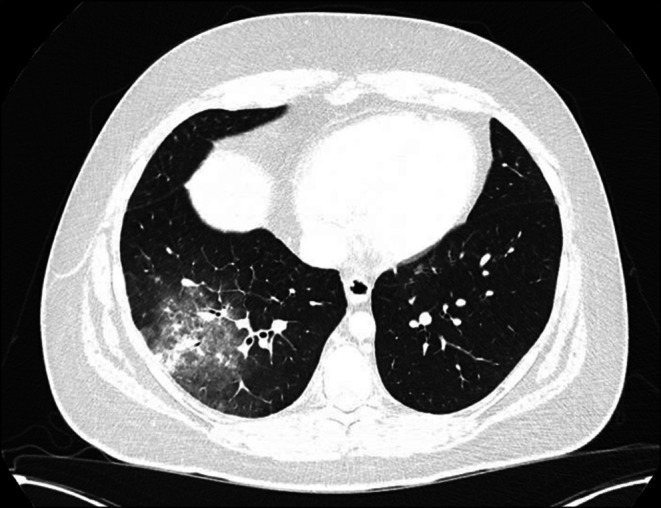
Transverse CT image of the thorax. Significant right lower lobe consolidation with ground‐glass opacification and interlobular septal thickening is visible.

A computed tomography (CT) study of the thorax, abdomen, and pelvis was requested, which revealed pulmonary consolidation in the right lower lobe suggestive of infection or inflammation (Figure 1). A small right‐sided pleural effusion and reactive adenopathy of the right hilar, subcarinal, and paratracheal nodes were also noted. No significant hepatosplenomegaly was present.

Bronchoalveolar lavage identified *Legionella pneumophila* and SARS‐CoV‐2, and was negative for acid‐fast bacilli and non‐tuberculous mycobacterium (NTM). Low‐level EBV reactivation was diagnosed during admission via peripheral blood qPCR testing (EBV DNA = 6,820 iU/mL, NR: Undetectable), with viraemia beginning to decline within a week and reaching < 1,000 IU/mL by week three.

**FIGURE 2:**
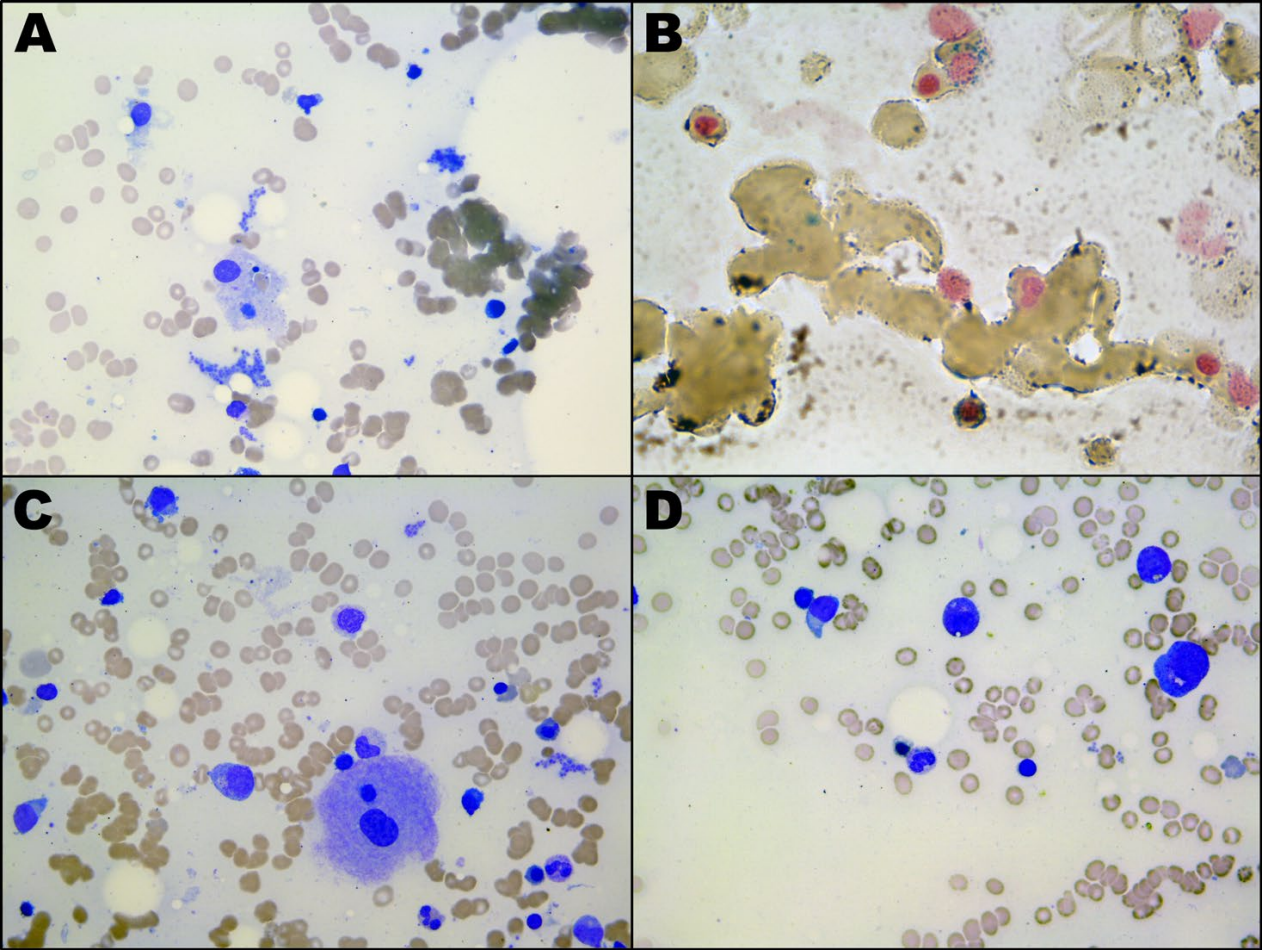
Photomicrographs of initial bone marrow aspirate. Hemophagocytosis (A), ring sideroblasts (B), dysplastic megakaryocytes (C), and dysplastic nucleated red cells (D) are visible.

A peripheral blood film appeared leukoerythroblastic and exhibited thrombocytosis, anisopoikilocytosis, and dacrocytes. Bone marrow aspirate and trephine were subsequently performed to investigate for underlying hematological abnormalities. Morphology revealed hemophagocytosis and red cell dysplasia, including the presence of ring sideroblasts (Figure 2). Flow cytometry revealed 10% CD34 positive blasts (CD45 weak, CD33 low); however, by morphology, blasts were not present in excess, consistent with MDS [21]. Next‐generation sequencing (NGS) and karyotyping of marrow samples were requested.

The patient's HScore was initially 161, but rapidly increased to 221 within four days (Table 2), indicating a 96%–98% probability of HLH [4]. Initial management was directed towards controlling hyperinflammation and treating underlying infection. Corticosteroids (dexamethasone 10 mg twice daily orally) and intravenous immunoglobulin (30 g daily for three days) were given. Oral clarithromycin (500 mg twice daily) and moxifloxacin (400 mg once daily) were adminstered for the treatment of *L. pneumophila* in accordance with local microbiological advice. Supportive treatments, including blood transfusion and electrolyte replacement, were prescribed as necessary throughout admission.

**TABLE 2:** HScore breakdown.

HScore Component	Result	Score
Known underlying immunosuppression	Absent	+0
Temperature (°C)	40.1	+49
Organomegaly	Absent	+0
Number of cytopenias	2	+24
Ferritin (mg/L)	9,358	+50
Triglycerides (mmol/L)	1.8	+44
Fibrinogen (g/L)	7.5	+0
Aspartate transaminase (U/L)	33	+19
Hemophagocytic features on bone marrow aspirate	Present	+35
Total	—	221

## 5 Definitive Investigation and Treatment

Twenty‐eight days after initial bone marrow biopsy, a second biopsy was performed to assess for evolving myeloid malignancy, identifying approximately 10% blasts by morphology and 16% by flow cytometry—consistent with MDS with increased blasts 2 (MDS‐IB2) and a high risk of progression to AML [21]. Karyotyping showed an abnormal clone exhibiting trisomy 8. NGS results indicated G2DS, revealing a *GATA2* variant (c.1154C > A (p.Pro385Gln)) with a variant allele frequency (VAF) of 49%, consistent with a heterozygous germline mutation. A somatic *SF3B1* variant (c.1998G > T (p.Lys666Asn), VAF = 29%) was also noted, with no other variants detected.

After discharge from the ward, the patient was referred to the regional HSCT service. Due to the progression to MDS‐IB2, one course of daunorubicin and cytarabine chemotherapy was given, resulting in complete morphological remission, although measurable residual disease was detected by flow cytometry at 0.377%. Remission bone marrow biopsy provided further evidence of the GATA2 variant's germline status, as VAF remained 49%. Allo‐HSCT (11/12 HLA‐matched unrelated donor) was then performed, following a conditioning regimen of FLAMSA (fludarabine, cytarabine, and amsacrine), total body irradiation, and antithymocyte globulin. Cyclophosphamide, mycophenolate mofetil, and ciclosporin were used for graft vs. host disease (GvHD) prophylaxis.

## 6 Outcome and Follow‐Up

The patient was monitored on the ward for four weeks post‐transplant. Recovery was complicated by successfully treated neutropenic sepsis, pulmonary emboli, and a small‐moderate abscess involving the inferior third of the left anal canal sphincter mechanism. Neutrophil and platelet engraftment were achieved on day nine. Immunosuppression was fully stopped after six months. Full (100%) donor chimerism in myeloid cells and T cells remained nine months post‐HSCT. No post‐transplant infection has occurred to date. Acute GvHD isolated to the skin (stage two) was successfully treated with a topical corticosteroid. Sanger sequencing of cultured skin fibroblasts confirmed the germline status of the *GATA2* variant, although family members have not yet been tested. He remains well without complications 15 months post‐transplant.

## 7 Discussion

HLH in G2DS has been reported previously but remains a rare entity [23]. Our case provides further evidence of the co‐occurrence of these conditions. It remains unclear whether G2DS conveys an independent genetic HLH risk via impaired immunoregulation or predisposes to it solely through an increased rate of precipitating atypical infections, although the latter is supported by all known cases occurring alongside infection [23]. Clinicians should aim to promptly diagnose G2DS when present in patients presenting with HLH to maximize the likelihood of correcting underlying hematological abnormalities secondary to G2DS and achieving sustained HLH remission [15, 24].

*GATA2* c.1154C>A (p.Pro385Gln) is a rare *GATA2* variant, currently classified as likely pathogenic (ClinVar accession: VCV001184200.1) [25]. The variant has been reported previously in a family of four first‐degree relatives, three of whom were symptomatic with features of G2DS, including MDS, T‐cell acute lymphoblastic leukaemia, atypical infections, DVT, and lymphoedema [26]. *GATA2* c.1154C > A (p.Pro385Gln) is a missense variant affecting the ZF2 domain, a region critical for the protein's DNA‐binding ability [15, 27]. Missense variants in the ZF2 domain comprise a significant proportion of pathogenic and likely pathogenic germline *GATA2* variants and are thought to alter GATA2 function via the disruption of DNA binding and protein–protein interactions [14]. Using the combined annotation‐dependent deletion (CADD) v1.7 model (GRCh38), the CADD‐PHRED score of this variant is 33, indicating that it lies among the 0.1% most deleterious predicted variants genome‐wide [28, 29]. The classification of *GATA2* c.1154C>A (p.Pro385Gln) may change over time as new evidence emerges.

This is the first documented case of an *SF3B1*‐mutated myeloid neoplasm associated with G2DS. *SF3B1* mutations are commonly found in MDS in the absence of G2DS, particularly when an excess of ring sideroblasts is present [22, 30]. This case represents a further example of a known MDS‐related mutation being described for the first time in G2DS, complementing existing knowledge of other variants associated with myeloid neoplasms which are well described in GATA2 deficiency, such as *ASXL1* and *STAG2* [20].

The positive outcome of allo‐HSCT in this patient provides further evidence of its potential efficacy for HLH in G2DS. Eight patients treated with allograft are reported in the literature, six of whom survived to the conclusion of follow‐up, which varied in length [23].

Our case exhibited several unusual characteristics, including thrombocytosis. Thrombocytopenia is a more common presentation of HLH, which has frequently been reported in HLH with underlying G2DS [1, 2, 23]. This finding illustrates the value of the HScore in aggregating the risk of HLH, rather than depending upon individual laboratory results.

Respiratory infection is well described in G2DS, with atypical pathogens such as NTM, *Aspergillus*, and *Histoplasma* often noted [11, 16]. The pathogens described in this case have previously been observed in G2DS [31, 32]. Prior cases of HLH in G2DS have been attributed to several organisms, including *M. avium* , *M. kansasii* , EBV, CMV, VZV, HSV, and influenza A, but not *L. pneumophila* and SARS‐CoV‐2, although these are recognized triggers of HLH in other individuals [23, 33, 34]. *L. pneumophila* is a Gram‐negative intracellular aerobic bacterium associated with a spectrum of disease ranging from a mild influenza‐like illness to the more severe legionnaire's disease, characterized by pneumonia [35]. *Legionella* infection becomes more common with advancing age but is also seen in those with compromised immunity, which may explain its occurrence in this young male with G2DS [36, 37]. SARS‐CoV‐2, a coronavirus, is the causative agent of COVID‐19, a respiratory infection that varies in severity from asymptomatic to life‐threatening [38]. Severe COVID‐19 infection, also more common in immunocompromised patients, can cause acute pneumonia with ground‐glass opacities and consolidation, as observed in this case [38, 39]. While EBV reactivation was detected, the relatively low level and short duration of EBV viraemia indicate that it was likely a reactivation associated with illness or steroid use, rather than a primary HLH trigger [40]. Our case, therefore, adds to the known list of pathogens that can precipitate HLH in patients with G2DS. Clinicians should note that a broad range of infections may trigger HLH in this population, and the list of reported organisms to date is likely far from exhaustive.

## 8 Conclusion

The occurrence of HLH in G2DS is rare, with only a small number of reported cases existing in the literature. The HLH risk in G2DS is likely due to predisposition towards precipitating atypical infections, combined with impaired immunoregulation. Clinicians should recognize and treat such patients promptly to maximize the likelihood of a positive outcome. An underlying diagnosis of G2DS should be excluded in all young adults presenting with bone marrow failure and atypical infection, including those with HLH. Where G2DS is confirmed, appropriate investigations should be performed to exclude associated hematological malignancies, such as myeloid neoplasms. Allo‐HSCT is indicated when life‐threatening complications of immunodeficiency, immune dysregulation, or acquisition of secondary clonal mutations in the bone marrow develop and should be considered in all eligible patients.

## Author Contributions

**Harry Wilson:** data curation, writing – original draft. **Nuno Borges:** data curation, writing – review and editing. **Matthew Collin:** data curation, writing – review and editing. **Callum Wright:** data curation, writing – review and editing.

## Funding

This work was supported by Blood Cancer UK (25014) and the Academy of Medical Sciences (SGCL033\1052).

## Consent

The patient provided informed written consent for the publication of this case report in accordance with journal policy.

## Conflicts of Interest

The authors declare no conflicts of interest.

## Data Availability

The data that support the findings of this study are available from the corresponding author upon reasonable request.
